# Direct Fabrication of Inclined-Sidewall Microgrooves with Shaped Flat-Top Beams

**DOI:** 10.3390/ma18225204

**Published:** 2025-11-17

**Authors:** Jianyong Mao, Wenqiang Chen, Kai Chen, Xun Li, Yu Tan, Ming Li, Lei Zhang

**Affiliations:** 1 Key Laboratory for Physical Electronics and Devices of the Ministry of Education & Shaanxi Key Laboratory of Information Photonic Technique, School of Electronic Science and Engineering, Xi’an Jiaotong University, Xi’an 710049, China; mjy2013@stu.xjtu.edu.cn (J.M.);; 2 State Key Laboratory of Ultrafast Optical Science and Technology, Xi’an Institute of Optics and Precision Mechanics of CAS, Xi’an 710119, China; chenkai@opt.ac.cn (K.C.);; 3 School of Mechanical Engineering, Shandong University, Jinan 250061, China

**Keywords:** microgroove, direct laser writing, flat-top beam, single-pass 3D ablation, beam-controlled morphology

## Abstract

Microgrooves have demonstrated significant application value across various fields. As a key processing technology, femtosecond laser processing exhibits notable advantages due to its high precision and minimal thermal impact. This study presents a method for fabricating microgrooves with inclined sidewalls using shaped flat-top laser beams. By controlling both the beam shape and scanning parameters, microgrooves with tailored sidewall morphology are produced in a single scan on silicon wafers. The optical performance of the fabricated structures is further evaluated through blazed grating prototypes. The experimental results aligned well with theoretical predictions. These results confirm that the proposed approach provides a viable technical pathway for efficient and high-quality fabrication of functional microstructures on comparable materials.

## 1. Introduction

Microgroove structures possess flexibly tunable and diverse configurations. Because of this, they are finding increasingly widespread applications in mechanics, biomedicine, optics and other fields [1]. In mechanical applications, surfaces textured with microgrooves effectively modulate component wettability, reduce friction, extend service life, and contribute to energy conservation, environmental protection, and desorption processes [2,3,4]. Within the medical domain, controlling the morphology of microgrooves on implant surfaces is crucial. It enables the regulation of key cellular behaviors, including adhesion, proliferation, migration, and differentiation [5,6,7,8,9]. Notably, microgrooves serve as fundamental building blocks for optical components such as diffraction gratings and Fresnel lenses. These components have important applications in information processing and spectral imaging. Furthermore, microgroove-induced shear stress can facilitate the alignment of liquid crystal molecules with superior switching characteristics and lower operating voltages compared to traditional polyimide layers [10,11,12,13].

Microgroove structures hold significant application value, yet their fabrication faces challenges in balancing efficiency, quality, and cost. Conventional techniques like mechanical machining often induce defects such as burrs, cracks, and high surface roughness. These defects stem from tool wear, residual stress, and inconsistent quality, especially on brittle materials and consequently compromise precision and consistency [14,15,16,17,18,19]. Chemical etching lacks anisotropy and employs corrosive agents [20,21,22], while high-precision methods like focused ion beam (FIB) and electron beam lithography (EBL) suffer from low throughput and high cost. Additionally, FIB can cause potential sputtering damage [23]. Femtosecond direct laser writing (DLW), known for its non-contact nature and flexibility, enables high-quality, thermal-damage-free processing [24,25,26,27,28,29,30]. However, efficient fabrication of complex groove geometries requires precise spatial energy control beyond the fixed Gaussian profile.

Both femtosecond DLW and mechanical machining are constrained by their inherent serial processing nature, which presents a significant bottleneck for large-scale microgroove fabrication. Furthermore, the layer-by-layer or point-by-point scanning strategies struggle to produce continuously smooth inclined sidewalls. In response to these challenges, previous research has demonstrated the potential of shaped femtosecond laser beams for single-scan fabrication of microgrooves. Meanwhile, conventional beam shaping using apertures or grayscale masks often leads to optical field inhomogeneity, defocus diffraction, and severe focal spot degradation, resulting in inefficient energy use and suboptimal shape control, particularly for high-aspect-ratio structures [31].

Building on our previous work, arbitrary flat-top beam shaping by reconstructing target beam amplitude distribution (RTAD) at the descending edge [32] can be realized to overcome the conflict between uniformity of the flat-top beam and severe energy loss using conventional phase retrieval algorithms. Adaptive optical elements, such as spatial light modulators (SLMs) and digital micromirror devices (DMDs), enable rapid switching between beam shapes. This capability greatly facilitates the programmable processing of customized structures. Furthermore, introducing an extra deflection or defocus phase can minimize the influence of the zero-order beam [33,34].

Here, we demonstrate an approach for fabricating microgrooves with inclined sidewalls using shaped flat-top laser beams. Through the judicious design of beam-shaping phases using the mixed-region amplitude freedom (MRAF) algorithm with RTAD, flat-top beams of various geometries and sizes can be generated. By controlling both the beam shape and scanning parameters (such as repetition rate, scan speed, etc.), microgrooves with tailored sidewall morphology can be produced in a single scan. Blazed gratings with varying periods and blaze angles are fabricated and their diffraction effects are further characterized to verify the effectiveness of this fabrication approach. The measured diffraction properties match well with the theoretical predictions. These results confirm that the proposed method provides a viable technical pathway for efficient and high-quality fabrication of functional microstructures.

## 2. Theoretical Model

### 2.1. Working Principle of Shaped Beam for Microgroove Fabrication

For DLW with discrete Gaussian beam pulses, the fabrication quality depends on several parameters, including the repetition rate, pulse energy, hatch distance, and scan speed. The structure relies on the specific deposition of energy enabled by mapping out suitable scanning paths and time intervals, a technique also known as temporal pulse shaping. The laser fluence distribution of a single tailored Gaussian pulse *F*(*x*,*y*,*z*) in the focal plane is described by [31].

(1) $$F\left(x,y,z\right)=\frac{2E_{p}}{\pi w_{0}^{2}}\exp\left(-\frac{2\left(x^{2}+y^{2}\right)}{w_{0}^{2}}\right)\delta \left(x,y,0\right)\times I_{ratio}\left(x,y,z\right)$$

where *E*_p_ is the pulse energy, *w*_0_ is the waist radius of the incident Gaussian beam and *δ*(*x*,*y*,*0*) is the window function of the designed mask. *I*_ratio_(*x*, *y*, *z*) is the deformation coefficient between the off-focal planes and the focal plane. This factor is particularly significant under an objective lens with a large numerical aperture (NA). In contrast, the flat-top beam generated by the RTAD method spans only a few hundred micrometers. This enables the implementation of a compact 4f beam reducing system utilizing a low NA objective lens. This configuration mitigates diffraction effects and preserves the *I_ratio_*(*x*, *y*, *z*) profile with negligible deviation over a defined distance near the focal plane. Additionally, beam homogenization transforms the initial Gaussian envelope into a uniform profile, allowing the laser energy fluence distribution to be effectively approximated by

(2) $$F\left(x,y,z\right)=\frac{E_{p}}{S}\delta \left(x,y,0\right),$$

where *S* stands for the area of the flat-top beam. According to the ablation threshold model for femtosecond laser machining [35], the etching depth *h*(*x*,*y*) of a single pulse along the *z*-axis of microgroove for single photon absorption is predicted as *h*(*x*,*y*) ≈ α^−1^ ln(*F*(*x*,*y*)/*F*_th_), where *α* is the material absorption coefficient and *F*th represents the ablation threshold of target materials for a single pulse.

The threshold fluence for multi-shot ablation, *F_th_*(*N*), is governed by the empirical relationship *F_th_*(*N*) = *F_th_N^T−1^* for silicon, glasses, and other brittle dielectrics. In this relation, *T* represents the slope of the incubation curve and typically ranges from 0.8 to 0.9 [36]. Consequently, the etching depth by a sequence of pulses along the *y*-axis, *h*(*y*), can be expressed as:

(3) $$h(y)=\sum_{n=1}^{N}\alpha^{-1}\ln\left(\frac{\frac{E_{p}}{S}\delta (x,y,0)}{\left(F_{th}\cdot n^{T-1}\right)}\right)=\frac{N}{\alpha }\ln\left(\frac{E_{p}\delta (x,y,0)}{S\cdot F_{th}}\right)+\frac{1-T}{\alpha }\ln(N!).$$

For *N* below 1000, the etching depth *h*(*y*) can be approximated as

(4) $$h(y)=\frac{1}{\alpha }\left[\ln\left(\frac{E_{p}\delta (x,y,0)}{S\cdot F_{th}}\right)+6.08\cdot (1-T)\right]\cdot N-331.03.$$

**Figure 1:**
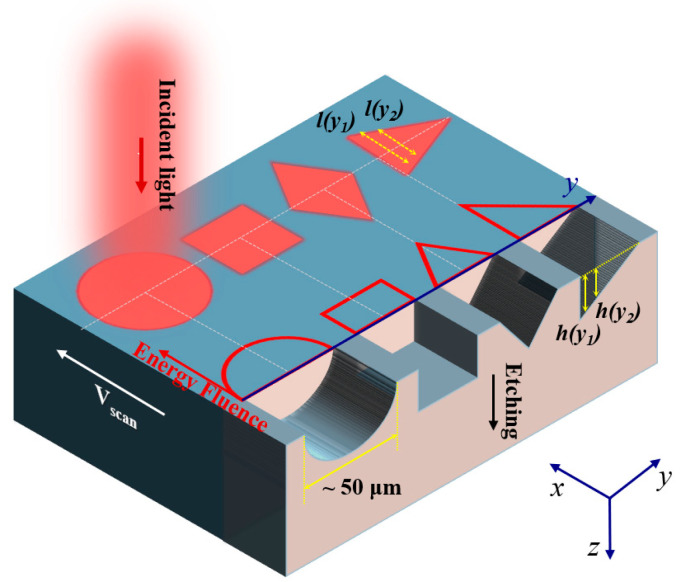
Schematic of microgroove fabrication using shaped laser beams.

This expression indicates a key relationship: the etching depth *h*(*y*) is approximately proportional to the effective pulse number *N*. *N* is calculated as $N\;=\;f\cdot l\left(y\right)/v$ under the condition of *F_th_* = 480 mJ/cm^2^ [37] and *α* = 30.2 cm^−1^ [38]. This parameter determines the laser energy fluence and is defined by the repetition frequency *f*, the beam length *l*(*y*) along the scanning direction, and the scan speed *v*. Based on this principle, the direct fabrication of microgrooves with designed cross-sections in a single step becomes possible. This is achieved by controlling the flat-top beam geometry, repetition frequency, scanning speed and pulse energy, as illustrated in Figure 1.

### 2.2. Optimization of Beam Shaping Phase

**Figure 2:**
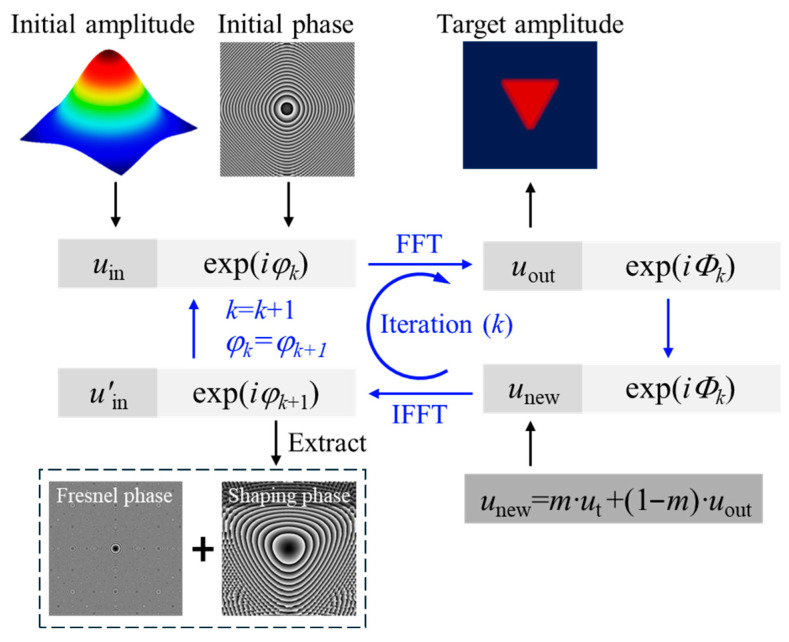
The flowchart of beam shaping phase design. FFT: Fast Fourier Transform; IFFT: Inverse Fast Fourier Transform.

It was demonstrated in our previous work that a smooth attenuation at the edge of the target beam profile effectively mitigates spectral leakage, enabling the generation of highly uniform, oscillation-free flat-top beams in a variety of shapes [32]. As illustrated in Figure 2, MRAF is employed as the core technique to achieve superior spot uniformity. The input field (*u_in_*), the reconstruction field (*u_new_*) and shaping phase (*φ_k+_*_1_) are designated to substitute $u_{in}^{\prime }$, *u_out_* and initial phase (*φ_k_*) in each iterative cycle, respectively.

The amplitude distribution of the incident Gaussian beam is given by

(5) $$u_{in}=Ae^{-\left(x^{2}+y^{2}\right)/w_{0}^{2}},$$

where *A* is the amplitude of the incident Gaussian beam (normalized to unity), and *x* and *y* denote the horizontal and vertical coordinates on the hologram plane, respectively. For enhanced convergence of the algorithm, a quadratic phase is employed as the initial condition, which is given by

(6) $$\varphi_{\mathrm{in}}=ax^{2}+by^{2},$$

where *a* and *b* are the coefficients for the horizontal (*x*) and vertical (*y*) coordinates on the hologram plane, respectively. Optimal speckle suppression can be achieved when the spatial frequency of the hologram reconstruction is within 0.5 to 2 times the bandwidth of the target illumination [39,40,41].

(7) $$a_{\min}=\frac{l\pi }{4hw_{0}\Delta x};\;a_{\max}=\frac{l\pi }{hw_{0}\Delta x}\;\mathrm{and}$$

(8) $$b_{\min}=\frac{l\pi }{4hw_{0}\Delta y};\;b_{\max}=\frac{l\pi }{hw_{0}\Delta y},$$

where *a*_min_ and *a*_max_ are the minimum and maximum coefficients, respectively, for the horizontal coordinate; while *b*_min_ and *b*_max_ are the corresponding values for the vertical coordinate; *l* is the separation between adjacent diffraction orders of the target flat-top beam; *h* is the width of the hologram. The parameters Δ*x* and Δ*y* represent the pixel size of the hologram along the *x* and *y*-axes, respectively. A key metric for evaluating the simulation results is the flat-top beam uniformity (γ), defined as

(9) $$\gamma =1-\sqrt{{\sum_{\left(\varepsilon ,\eta \right)\in W}\left[\frac{I\left(\varepsilon ,\eta \right)-\bar{I}}{\bar{I}}\right]}^{2}/n-1},$$

where *W* is the area of the flat-top beam at the reconstruction plane; *I*(ε, *η*) is the intensity distribution at the reconstruction plane; $\bar{I}$ is the mean intensity within *W*; *n* is the total number of discrete sampling points within the region. The uniformity factor (*γ*) serves as a quantitative measure of the uniformity within the target area. A value of *γ* closer to 1 thus indicates a more ideal flat-top beam profile.

Furthermore, it is critical to mitigate undesirable etching effects caused by the zero-order diffraction beam. Conventional beam shaping configurations employ a lens to perform Fourier transformation for generating a flat-top beam. The pixelated nature of the SLM, however, differentiates its active area into signal and non-signal regions. Unmodulated reflective light from the non-signal regions is focused by the lens, leading to the appearance of a zero-order beam. This work addresses this issue by substituting the physical lens with a Fresnel lens phase pattern as shown in Figure 2. By implementing a lens phase with equivalent discretization, unmodulated reflective light from non-signal regions is not modulated by the Fresnel lens phase, thereby eliminating the formation of the zero-order beam.

## 3. Experimental Setup

**Figure 3:**
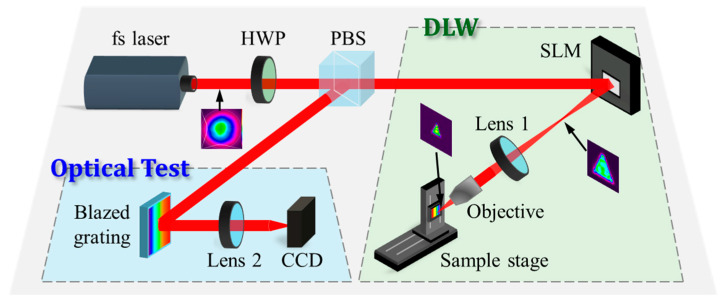
Experimental setup for DLW and optical diffraction test. Inset shows the triangular flat-top beam as an example for fabrication.

As illustrated in Figure 3, the DLW system is based on a commercial titanium-sapphire femtosecond laser (PH1-20-0400-10-30, Light Conversion, Vilnius, Lithuania) operating at a wavelength of 1030 nm, with a beam radius of 2.1 mm, *M*^2^ of 1.1, a pulse duration of 250 fs, and a base repetition rate of 50 kHz. A half-wave plate (HWP) is used to control the polarization angle of the incident light, and a polarization beam splitter (PBS) subsequently divides the beam into two paths: one for DLW and the other for optical characterization. A high-resolution beam profiler (Ophir SP932U, Ophir Optronics Solutions, Logan, UT, USA; resolution: 1536 × 2048, pixel size: 3.45 μm) is employed to capture both the initial Gaussian profile and the shaped beam profiles. The Gaussian beam is incident on the SLM at a small angle of 9°, as specified by the manufacturer, to ensure accurate phase modulation. The reflected beam, which carries the combined focusing and shaping phase information, forms a flat-top beam measuring several hundred micrometers at 100 mm. This beam is then further reduced to less than 50 μm using a compact 4*f* telescope system composed of a 125 mm lens and a 20× objective lens (effective focal length f = 10 mm, Evenoptics, Shanghai, China). Sample positioning in the *x*–*z* plane is controlled by a two-axis motorized stage (OSMS20-35, Optosigma, Changchun, China). The overall energy efficiency of the system is defined as the ratio of the power measured after the final shaped profile to that of the incident Gaussian beam before the SLM. To characterize the optical performance of the blazed grating, 1% of the incident beam power is split via the HWP and PBS and directed onto the grating at an angle of 5°. The diffracted beam is focused by a 125 mm focal length lens, and the resulting diffraction order distribution is captured by the beam profiler placed at the focal plane on an *x*–*y* stage.

The silicon wafers were mounted on the sample stage at the focal plane of the objective lens. A custom two-axis mount allowed for fine adjustment of the wafer’s tip and tilt, ensuring it remained within the focal plane during translation. Throughout the machining process, a stream of compressed air at 0.55 MPa was directed across the sample surface to remove generated residues and debris. Following processing, the samples underwent an additional 10 min ultrasonic cleaning in a 5% NaOH solution to decompose any remaining contaminants. The resulting surface topography was characterized using a confocal microscope (VK-X3000, Keyence, Shanghai, China).

Phase profiles for generating various flat-top beams—a 500 μm diameter circle, a 500 μm side-length square, and a 500 μm height triangle—were computed using the phase optimization algorithm described previously. Each calculated phase profile was loaded onto the SLM and combined with a Fresnel lens phase corresponding to a 100 mm focal length, projecting the shaped beam at the 100 mm plane. These beams were then collimated and demagnified using a 125 mm focal length lens and a 10 mm effective focal length objective, yielding final beam dimensions of a 40 μm diameter circle, a 40 μm side-length square (which could be rotated by 45° to produce a diamond profile), and a 40 μm height triangle. The experimentally measured uniformity of all generated flat-top beams exceeded 90%. These optimized beams were subsequently employed for microgroove fabrication.

## 4. Results and Discussion

**Figure 4:**
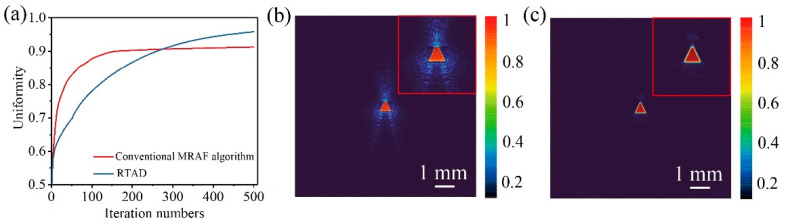
Comparison of beam shaping algorithms. (**a**) Comparison of conventional MRAF algorithm and RTAD; The normalized intensity distribution obtained using (**b**) conventional MRAF algorithm and (**c**) RTAD. Insets show the zoom-in intensity distributions.

To fabricate microgrooves with inclined sidewalls, a Gaussian beam was first shaped into various flat-top profiles. As illustrated in Figure 4 using a triangular flat-top beam as an example, the proposed RTAD method is compared against the conventional MRAF process. Figure 4a shows that, despite its slower initial convergence, the RTAD strategy achieves superior homogeneity after 500 iterations. We selected this iteration count because it reliably meets the uniformity requirement (>90%) for fabrication. Beyond this point, further iterations yield negligible improvement in beam quality while increasing computational cost, as indicated by the clear convergence trend. Given this asymptotic behavior, a fixed iteration count was employed as the stopping criterion instead of a threshold on uniformity. Notably, Figure 4b,c demonstrate that the conventional MRAF algorithm produces substantial speckle noise in the non-signal region. In contrast, the RTAD approach effectively minimizes such interference by engineering a smooth descent at the beam edge.

**Figure 5:**
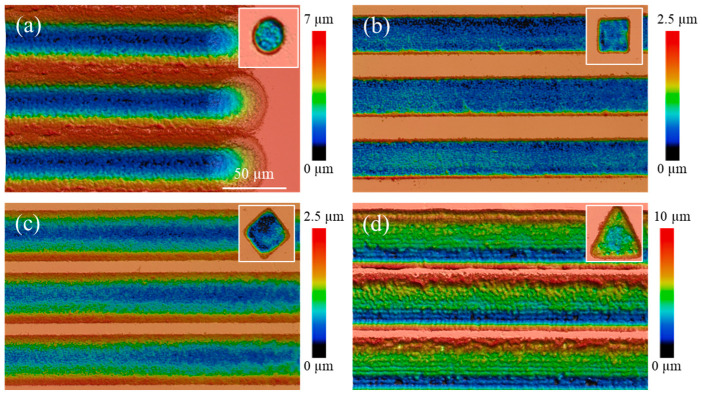
Height map of microgrooves fabricated using (**a**) circular (diameter 40 μm), (**b**) square (length 40 μm), (**c**) diamond (side length 40 μm) and (**d**) equilateral triangular (height 50 μm) shaped flat-top beam.

Using a pulse energy of 3.6 μJ and a pulse width of 250 fs, we first fabricated flat-bottomed microstructures by delivering 100 pulses per point. The resulting circular, square, diamond, and triangular shapes are shown in the insets of Figure 5. Subsequently, we employed the same shaped flat-top beams in a single-pass scanning mode at 50 kHz and 2 mm/s to produce continuous microgrooves. Their cross-sections corresponding to semi-elliptical, rectangular, equilateral triangular, and right-angled triangular geometries, are presented in Figure 5. These experimental results align well with the predictions in Figure 1.

For a blazed grating with a groove angle of θ controlled by varying the depth of sidewalls of structures and period, the condition for constructive interference at normal incidence is given by Psin(2θ)=mλ, where *P* is the grating period; *m* is the diffraction order; λ is the wavelength. Under the small-angle approximation, the corresponding grating depth of blazed grating is defined as

(10) $$H=P\tan\left(\theta \right)=P\tan\left(\frac{1}{2}\arcsin\left(\frac{m\lambda }{P}\right)\right)\approx P\cdot \frac{m\lambda }{2P}=\frac{m\lambda }{2}$$

For target periods (*P*) of 50, 45, and 40 μm, the first-order diffraction angles for 1030 nm incident light are calculated as 1.18°, 1.31°, and 1.47°, respectively, all requiring a grating depth of approximately 0.52 μm.

In the experiment, equilateral triangular flat-top beams were scanned along their bases on a polished (100)-oriented single-crystal silicon wafer. The height of each equilateral triangle was designed to match the target period of the corresponding grating. The scanning was performed at a repetition rate of 6.25 kHz and a speed of 5 mm/s, with the line spacing set equal to the target period for each grating.

**Figure 6:**
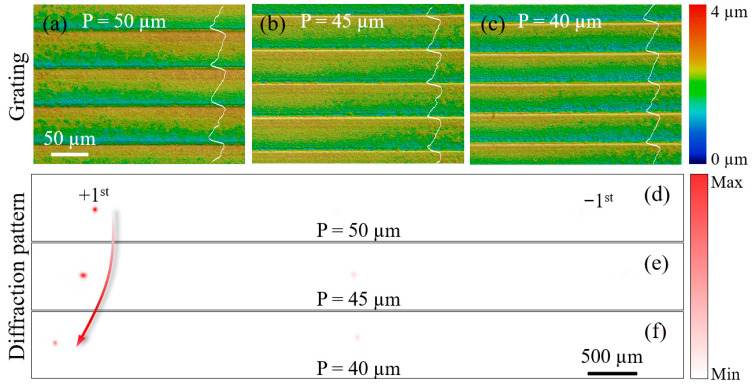
Diffraction of blazed gratings with varying periods. Height map of fabricated blazed grating with period (**a**) 50, (**b**) 45 and (**c**) 40 μm; Diffraction pattern of blazed grating with period (**d**) 50, (**e**) 45 and (**f**) 40 μm.

As summarized in Table 1, three equilateral triangular flat-top beams with designed heights of 50, 45, and 40 μm were used to fabricate blazed gratings. The measured grating periods were 49.9, 45.8, and 39.2 μm, respectively, as illustrated in Figure 6a–c. Based on an orthogonal experimental design, the pulse energy was attenuated to 4.48, 3.62, and 2.94 μJ (from a source output of 1.2 W), resulting in grating depths of 0.56, 0.57, and 0.55 μm, respectively. The corresponding diffraction patterns are presented in Figure 6d–f. The measured separations between the first order and 0-order diffraction spots are 2.68, 2.85, and 3.28 mm, corresponding to diffraction angles of 1.22°, 1.31°, and 1.50°, respectively. These values closely match the theoretical predictions, validating the designed grating periods. The slight deviations observed are primarily attributed to surface irregularities on the grating structures, likely caused by the residual inhomogeneity of flat-top beam and redeposited ablation debris that was not fully removed by the gas blowing process. This indicates that the current gas-assisted cleaning method, while beneficial, is insufficient to completely mitigate debris-related defects, thereby imposing a limitation on the achievable surface quality in precision microfabrication.

The use of shaped flat-top spots for microgroove processing enables precise control over the local energy deposition and, consequently, the etching depth at positions transverse to the scan direction. This is achieved by modulating the effective pulse number by either varying the line length intersected by the scanning path along the shape boundary or by adjusting the scan speed and repetition rate. In the context of blazed gratings, the etching depth, which can be accurately programmed by altering the base length of the triangular spot or the scanning velocity, determines the blaze angle and thus the diffraction efficiency into specific order.

Taking a blazed grating with a 40 μm period as an example, the theoretical blaze angles (θ) for the +1, +2, and +3 orders are 1.47°, 2.95°, and 4.43°, requiring grating depths of 0.52, 1.03, and 1.54 μm, respectively. An orthogonal experiment was conducted for this design, employing an equilateral triangular spot (height: 40 μm, pulse energy: 2.94 μJ, repetition rate: 5 kHz) at different scan speeds. The resulting grating depths and their corresponding performance are summarized in Table 2.

**Figure 7:**
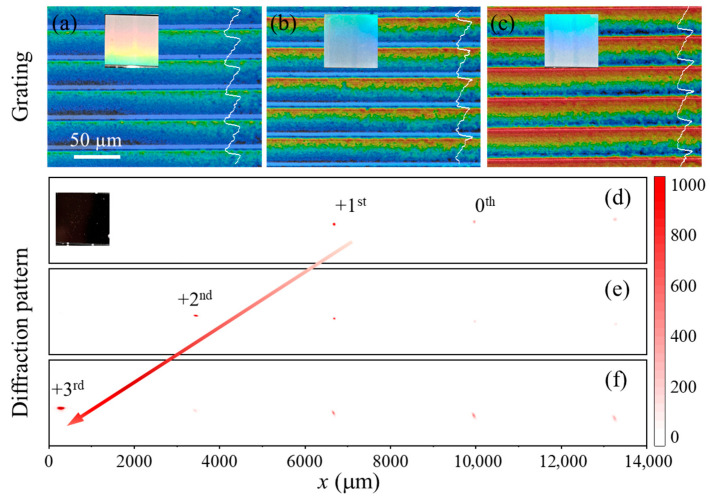
Diffraction of blazed gratings with varying sidewall angle and a fixed period 40 μm. Height map of fabricated grating with depth (**a**) 0.52 μm, (**b**) 1.03 μm, (**c**) 1.54 μm with insets showing corresponding their optical photos; Diffraction pattern of blazed grating with inclined angle (**d**) 0.74°, (**e**) 1.47° and (**f**) 2.20° with inset in (**d**) showing the optical photo of an unpatterned silicon wafer.

As shown in Figure 7a–c, the fabricated blazed gratings exhibited measured depths closely matching the design values. The insets in Figure 7 show optical micrographs of the respective grating structures. Diffraction pattern analysis using a 125 mm focal length lens confirmed that the primary diffracted orders were +1, +2, and +3, with measured diffraction angles aligning well with the designed blaze angles, as evidenced in Figure 7d–f.

Processing efficiency is a critical factor in scaling up the fabrication of microgroove structures. For instance, conventional Gaussian beam scanning would require approximately 29 passes to clear a single layer across a 40 μm-wide region. To achieve a 0.52 μm depth with a smooth inclined sidewall using a “serpentine + adaptive hatch” strategy—with a minimal depth increment of 0.1 μm per layer—the cumulative number of passes increases significantly to 76. This estimate is based on a diffraction-limited spot size of ~2.75 μm, derived from the experimental setup with the shaping phase removed and only the Fresnel lens phase (*f* = 100 mm) applied, and a 50% line overlap, which is a standard empirical value for line-by-line scanning. Such a multi-pass process severely limits throughput. In stark contrast, our approach using a shape-tunable flat-top beam achieves equivalent or superior groove geometry in just a single scan. This approach not only drastically enhances processing efficiency but also produces high-quality inclined surfaces without stair-step artifacts. Furthermore, the method offers exceptional controllability, enabling independent adjustment of groove width and sidewall angle through beam geometry, scan speed and pulse energy.

## 5. Conclusions

In conclusion, this study demonstrates a single-pass 3D ablation approach for fabricating microgrooves with inclined sidewalls on silicon wafers using shaped flat-top femtosecond laser beams. Through optimized beam-shaping phases, flat-top beams of various geometries were generated with a uniformity exceeding 90%. By controlling both the beam shape and scanning parameters, microgrooves approximately 40 μm wide with tailored sidewall morphology were produced in a single scan, representing an approximately 76-fold improvement in processing efficiency compared to the multi-pass “serpentine + adaptive hatch” strategy. The functionality of this approach was verified through blazed gratings with varied periods and blaze angles, whose measured diffraction properties agreed well with theoretical predictions, exhibiting depth errors below 10% and diffraction angle deviations within 0.1°. These results confirm the feasibility of the proposed method in fabricating functional microgroove structures on silicon and similar brittle materials. Future work will focus on in situ process monitoring and extension of this technique to other functional materials.

## Figures and Tables

**Table 1:** Experimental parameters and fabricated grating characteristics.

Period (μm)		Pulse Energy (μJ)	Grating Depth (μm)		Diffraction Angle (°)		Deviation from Theory
Designed	Measured		Designed	Measured	Designed	Measured	
50	49.9	4.48	0.52	0.56	1.18	1.22	3%
45	45.8	3.62		0.57	1.31	1.31	0%
40	39.2	2.94		0.55	1.47	1.5	2%

**Table 2:** Design and experimental results for 40 μm period blazed gratings.

Target Diffraction Order	Diffraction Angle (°)		Scan Speed (mm/s)	Grating Depth (μm)	
	Designed	Measured		Designed	Measured
1	1.47	1.48	6	0.52	0.53
2	2.95	2.95	3	1.03	1.03
3	4.43	4.52	2	1.54	1.58

## Data Availability

The original contributions presented in this study are included in the article. Further inquiries can be directed at the corresponding authors.
